# Digital phenotyping for monitoring and prediction of physical activity level during pregnancy

**DOI:** 10.1093/eurpub/ckac131.168

**Published:** 2022-10-25

**Authors:** M Shojaa, N Schmitz

**Affiliations:** Population-Based Medicine, Tuebingen University, Tuebingen, Germany

## Abstract

**Background:**

Gestational diabetes mellitus (GDM) is one of the most common complications in pregnancy. Physical activity (PA) during pregnancy may reduce the adverse pregnancy outcomes. Digital phenotyping can develop interactive risk profiles to better reflect a person’s daily mobility than traditional retrospective self-assessment questionnaires. Therefore, the aims of the proposed research are to 1) test the acceptability and applicability of the BEIWE app during pregnancy, 2) characterize daily physical mobility during pregnancy, and 3) describe differences in physical mobility in women with and without diabetes.

**Methods:**

This prospective cohort study will include 20 pregnant women with GDM and 20 without GDM from the Diabetes Center of the Medical Clinic or outpatient clinics of the Tübingen Women’s Clinic, Tuebingen, Germany. The study was approved by the Ethics Committee of the University Hospital Tuebingen (004/2022BO1). At baseline (22 weeks gestation), participants will be instructed to download the app. Passive data (phenotypic information) is collected automatically during the observation period. The follow-up assessment will be conducted three weeks after delivery. The acceptance of the app and comparison of the groups with and without diabetes will be conducted through Statistical analysis.

**Results:**

Recruitment of participants has started and follow-up assessment is estimated to be finalized in fall 2022. Study design and results will be presented at the conference.

**Conclusions:**

PA during pregnancy has been associated with minimum risk of a pregnancy, and self-monitoring of PA via an app may play a role in improving pregnancy outcomes. However, the success of Apps depends on their validity and reliability, which lack evidence.

**Key messages:**

• The result of the study will develop a qualification measure for PA with no intervention.

• It also provides information for planning and conducting subsequent intervention studies.

